# Comparison of NaOH-Based Solvents for Lignocellulosic Microfines (LCMFs) Dissolution and Properties of Regenerated Cellulose Film

**DOI:** 10.3390/gels12030199

**Published:** 2026-02-27

**Authors:** Jiae Ryu, Sa Rang Choi, Jung Myoung Lee

**Affiliations:** Department of Wood and Paper Science, Kyungpook National University, 80 Daehakro, Daegu 41566, Republic of Korea; wldo1995@gmail.com (J.R.); luvvchoi@knu.ac.kr (S.R.C.)

**Keywords:** cellulose dissolution, regenerated cellulose (RC), lignocellulosic microfines (LCMFs)

## Abstract

Cellulose dissolution solvents have been developed for the fabrication of regenerated cellulose (RC) films, which are known for their high optical transparency, excellent barrier properties, and biodegradability. In this study, three types of aqueous dissolution systems, including glycol ether/sodium hydroxide (NaOH), poly(ethylene glycol) (PEG)/NaOH, and urea/NaOH aqueous systems, were investigated to compare their effects on lignocellulosic microfine (LCMF) solutions and the resulting regenerated cellulose films. The dissolution yields of LCMFs in these solvents ranged from 77.0% to 85.0%. The incorporation of glycol-based co-solvents in NaOH significantly influenced the transparency (over 70% of transparency) of the regenerated LCMF films. The use of a high molecular weight of co-solvent (PEG) especially resulted in enhanced stability of the LCMF solutions, as evidenced by higher inherent viscosities and the minimal viscosity change over 30 days compared to glycol ether/NaOH and urea/NaOH systems. Furthermore, the films regenerated from the PEG/NaOH solvent showed the lowest shrinkage (19.4%) and the highest mechanical strength (47.8 MPa), followed by the glycol ether/NaOH and urea/NaOH systems. These results confirm that the type of co-solvent in cellulose dissolution systems influences the composition, coagulation behavior, and drying characteristics of regenerated LCMF films, affecting their mechanical performance. This study provides insights into the effective utilization of lignocellulosic materials for the efficient fabrication of regenerated cellulose.

## 1. Introduction

Reducing non-renewable resource consumption and greenhouse gas emissions has been identified as an urgent global challenge [1]. In accordance with the Intergovernmental Panel on Climate Change (IPCC) climate targets, the rapid acceleration of commercial CO_2_ capture, utilization, and storage (CCUS) operations has been deemed necessary [2]. Within this context, woody biomass has been recognized as a more environmentally sustainable alternative to fossil-based products and as a valuable resource for cradle-to-grave circular systems [1,3,4,5,6]. Regenerated cellulose (RC), which derives from cellulose accounting for approximately 40–50% of woody biomass, has been utilized in diverse forms, including filaments, films/membranes, microspheres/beads, hydrogels/aerogels, and bioplastics [7,8,9,10]. Because of its renewable origin and environmental advantages, RC has attracted increasing interest as an alternative to conventional synthetic polymers.

RC is typically prepared by dissolution of cellulose chains in a suitable solvent, followed by coagulation. During this dissolution–regeneration process, the original supramolecular structure of cellulose is disrupted and subsequently reorganized. As a result, distinct crystalline structures of native cellulose (cellulose I) and regenerated cellulose (cellulose II) have been attributed to differences in hydrogen bonding patterns: parallel chain orientation has been associated with cellulose I, whereas antiparallel orientation has been associated with cellulose II, resulting in fewer voids and higher crystallinity than fiber-derived films [11,12,13,14]. As a consequence, RC films often exhibit high optical transparency, excellent barrier properties, and biodegradability [15]. These advantageous properties have motivated efforts to optimize RC film performance through processing control [13,16]. Nevertheless, persistent challenges arise from the intrinsic insolubility of cellulose in water and common organic solvents, attributed to its high crystallinity and extensive hydrogen bonding [17,18].

To address these limitations, numerous solvent systems have been developed to overcome cellulose dissolution recalcitrance and to enable RC film preparation [19]. It has been reported that cellulose solutions in certain alkaline or organic solvent systems exhibit time- and temperature-dependent gelation behavior during dissolution and storage, in which the polymer-rich phase shows increasingly restricted chain mobility and a viscoelastic response characteristic of a gel-like state, rather than a simple low-viscosity solution [20]. Cellulose solvents have been commonly classified as “derivatizing” or “non-derivatizing” systems [21]. In derivatizing systems, dissolution has been achieved through covalent modification via an unstable ether, ester, or acetal intermediate. This was an approach historically employed in the production of cellulose nitrate and viscose [22]. In contrast, dissolution in non-derivatizing systems has been achieved solely through physical intermolecular interactions [21], including non-aqueous solvents, ionic liquids, salt solutions, and alkali-based aqueous media [23,24,25].

Among non-derivatizing systems, NaOH-based aqueous solvents have been considered environmentally compatible, easy to handle, and cost-effective [26]. Accordingly, NaOH-based systems have attracted sustained attention as practical solvents for RC production. Cellulose with a low degree of polymerization can be easily dissolved at low temperature in a 7–10% NaOH solution. Therefore, several investigations on NaOH-based aqueous solutions have been conducted to produce dissolved and regenerated cellulose with a high degree of polymerization [27,28,29]. The urea/NaOH aqueous solution can dissolve 90% or more of cellulose or cellulose containing < 10% lignin, with a degree of polymerization (DP) of approximately 500, through a pretreatment process for dissolving the raw material. Urea/NaOH is a dissolving solvent used for the production of RC, and has been reviewed as part of NaOH-based aqueous solutions [30]. The PEG/NaOH aqueous solution, another dissolving solvent based on the NaOH aqueous solution, is a new dissolution solvent in which the dissolved cellulose solution remains stable. This solvent has been successfully used in the manufacturing of all-cellulose composites [28,31].

Despite extensive studies on NaOH-based aqueous systems for cellulose, most previous work has focused primarily on dissolution mechanisms, solubility enhancement, or the dissolution yield of purified cellulose [26,32,33,34]. Unlike the purified cellulose systems typically studied, LCMFs contain residual lignin and hemicellulose, which introduce additional intermolecular interactions and heterogeneity during dissolution and regeneration. This complexity makes it crucial to understand the gel behavior, phase stability, and network formation that occur during LCMF dissolution. However, the comparative effects of different NaOH-based co-solvents on the stability of lignocellulosic solutions, coagulation behavior, drying-induced structural evolution, and the resulting film properties have not yet been discussed. Moreover, lignocellulosic microfines (LCMFs), which retain residual lignin and hemicellulose, do not behave in the same manner as purified cellulose during alkaline dissolution and regeneration. These non-cellulosic components can interfere with cellulose chain disentanglement and re-association in NaOH-based systems, thereby affecting solution stability and the formation of a continuous regenerated network [35,36]. Nevertheless, how these effects vary with the type of cosolvent in NaOH-based aqueous systems has not been clearly examined.

Therefore, the LCMF feedstock [37], prepared via an organosolv pulping process with controlled residual lignin content through sequential washing treatments, was employed to compare glycol ether/NaOH, PEG/NaOH, and urea/NaOH solvent systems and to evaluate their effects on dissolution behavior and regenerated film properties.

## 2. Results and Discussion

### 2.1. Dissolution Characteristics of Dissolved and Undissolved Components

**Figure 1:**
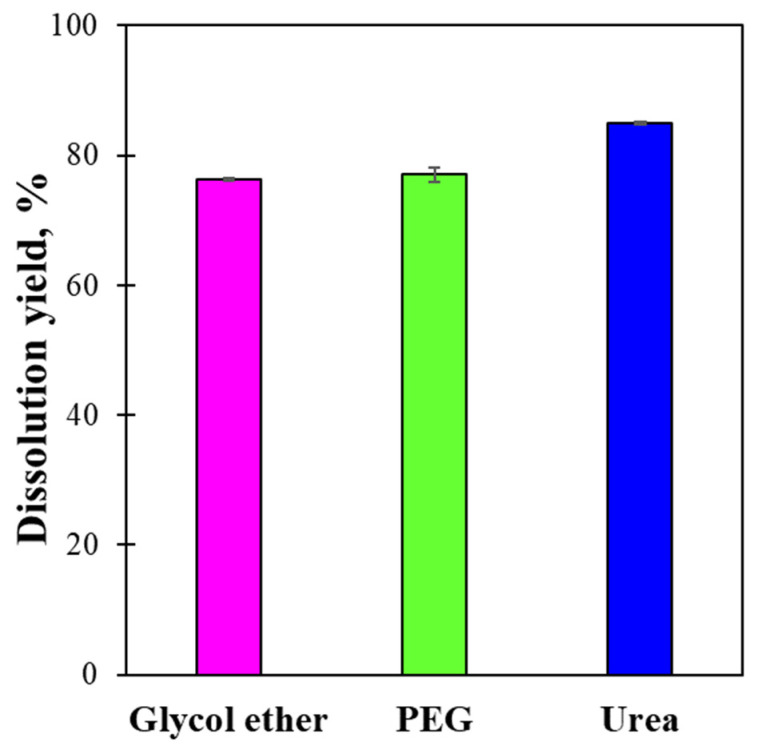
Comparison of dissolution yields of LCMFs in different dissolution solvents.

The dissolution yields of LCMFs are shown in Figure 1. Although the degree of LCMF polymerization was 1250, the urea/NaOH solvent exhibited the highest dissolution yield (85.0%), followed by the PEG/NaOH (77.0%) and glycol ether/NaOH (76.2%) solvents. In NaOH aqueous solutions, NaOH disrupts the hydrogen bonds between cellulose molecules. Here, urea has an amphiphilic structure, influencing the cellulose dissolution [33]. The hydrophobic part of urea interacts with the hydrophobic part of the cleaved cellulose. The hydrophilic part of urea forms a hydrogen bond with water, ensuring that the dissolved cellulose chains remain well separated in the liquid media. Therefore, the additional urea resulted in higher dissolution yields than the neat NaOH aqueous solution. In addition, the freeze–thaw process has been reported to improve cellulose dissolution yield [38]. Glycol ether/NaOH and PEG/NaOH exhibited a lower dissolution yield than the urea/NaOH solvent, but still comparable. Here, the co-solvents act as a blocker in NaOH aqueous solutions. This stabilizes the solution by preventing the re-aggregation of hydrogen bonds destroyed by the NaOH aqueous solution [26].

Table 1 and Table 2 summarize the mass distribution of raw LCMFs and the corresponding dissolved and undissolved fractions after dissolution using different NaOH-based solvents. While overall dissolution yields varied across the solvent systems, the mass balance analysis in Table 2 confirms that these differences directly reflect the relative amounts of regenerated and residual components. Compared to the dissolved and undissolved components, the major dissolved component through the NaOH-based solvents was carbohydrates. The urea/NaOH solvent dissolved the highest carbohydrates (71.6 g), followed by PEG/NaOH (68.2 g of carbohydrates), and glycol ether/NaOH (67.7 g of carbohydrates) solvents. Lignin in LCMFs also dissolved in the NaOH-based solvent, showing 5.2 g lignin dissolution from the urea/NaOH solvent, compared to 3.9 g and 3.5 g of lignin from PEG/NaOH and glycol ether/NaOH solvents, respectively. There was only 12 g of carbohydrate and 2.3 g of lignin in the undissolved component from the urea/NaOH solvent. The results demonstrate that LCMFs have a higher dissolution yield with the urea/NaOH solvent, whereas the PEG/NaOH and glycol ether/NaOH solvents show comparable dissolution levels.

Table 3 shows the Klason lignin content and neutral sugar analysis according to the dissolved raw material and solvent. The most obvious difference in the dissolution process can be observed between the residual lignin content of the dissolved (regenerated film) and undissolved (unreacted residue) components. The dissolved component had about half the residual lignin content of the raw material (8.4%), whereas the undissolved component had about twice the residual lignin content. These results show that the lignin dissolution has a limit that depends on the type of dissolution solvent used. The urea/NaOH solvent dissolved the highest lignin content. Dissolution yield was also the highest for the urea/NaOH solvent, and it was found that carbohydrates as well as lignin were quantitatively dissolved. As a result of the neutral sugar analysis, the hemicellulose content was found to be the highest in the dissolved component. This indicates that hemicellulose is more soluble than cellulose. The dissolution of hemicellulose, such as xylose and mannose, by an aqueous-alkali solution has been reported in previous work [30]. However, no significant difference was found in the carbohydrate compositions for the three types of dissolution solvents. These results confirm that the factors influencing the dissolution yield of the dissolution solvent are determined by the dissolution degree of the overall carbohydrates and lignin.

### 2.2. Properties of the Dissolved LCMF Solution

**Figure 2:**
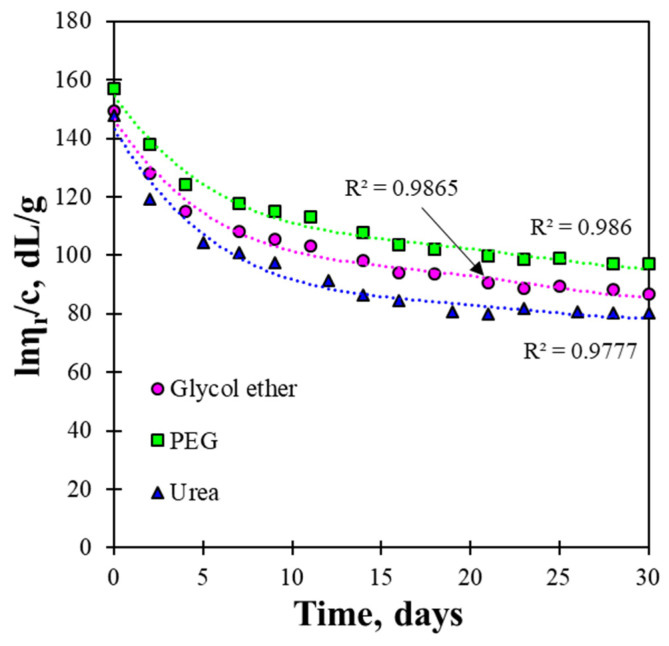
Inherent viscosity of LCMF solutions prepared using different dissolution solvents.

The stability of the cellulose solution is an important factor in regenerated film manufacturing and can be effectively evaluated by monitoring inherent viscosity, which reflects changes in molecular integrity during storage. After dissolving LCMFs with different solvents, the inherent viscosity of the LCMF solution was measured over 30 days (Figure 2). The viscosity reduction may be associated with gradual structural rearrangement or limited alkaline degradation of cellulose chains under NaOH-based conditions, as reported in previous studies [26].

The inherent viscosity decreased over the entire storage time. The fresh LCMF solution using the PEG/NaOH solution (157.14 dL/g) was slightly higher than that of the other two solvents, followed by LCMF-glycol ether/NaOH and LCMF-urea/NaOH solutions. The LCMF-urea/NaOH solution’s inherent viscosity was similar to that reported in a previous study using a 6 wt% NaOH/4 wt% urea aqueous solution (about 150 dL/g at 25 °C) [38]. As storage time increased, the inherent viscosity decreased for all solvent systems. The reduction was most pronounced for the urea/NaOH solvent, followed by the glycol ether/NaOH solvent and the PEG/NaOH solvent. The reduction amounts were 68.7, 63.3, and 62.0 dL/g for the urea/NaOH, glycol ether/NaOH, and PEG/NaOH solvents, respectively. It was reported that the stability of the cellulose solution is affected by the degree of recombination of the cellulose chains and is maintained as the temperature decreases; however, the cellulose solution becomes unstable as the temperature increases [38,39]. Likewise, the stability of the cellulose solution over storage time is also significant for investigating the cellulose chain arrangement and comparing the effects of co-solvent solutions. At room temperature (25 °C), we observed that the decrease in inherent viscosity was lowest in the LCMF-PEG/NaOH solution, indicating that cellulose and lignin chains were most stably maintained by the PEG/NaOH solvent. The PEG used in the dissolving solvent has a long polymer chain with a molecular weight of 2000, which is considered a key factor in preventing recombination between cellulose molecules over time. Therefore, the PEG/NaOH solvent has provided the most stable cellulose arrangement with comparable dissolution ability, compared to the glycol ether/NaOH and urea/NaOH solvents.

**Figure 3:**
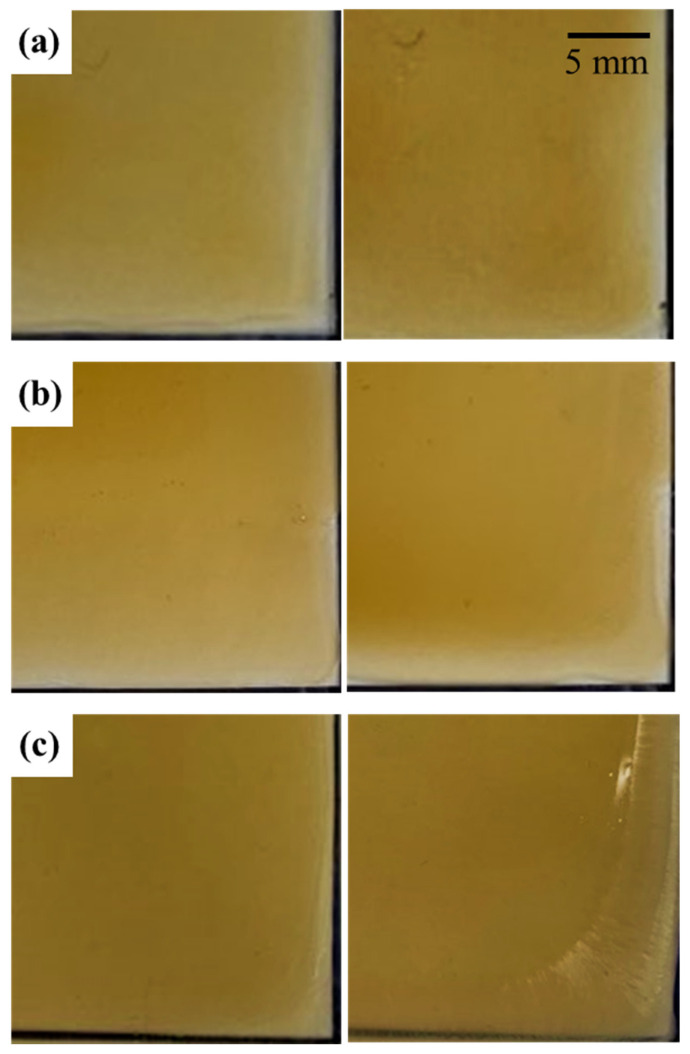
Dried LCMF solution (**left**: immediately after casting, **right**: completely dried) for (**a**) glycol ether/NaOH solvent, (**b**) PEG/NaOH solvent, and (**c**) urea/NaOH solvent.

During the drying process, solvent stability is crucial for preserving dissolved lignocellulosic constituents. After dissolution, the LCMF solutions were cast onto a glass plate to form an LCMF film and dried (Figure 3). As shown in Figure 3a,b, the LCMF solutions prepared with the glycol ether/NaOH and PEG/NaOH solvents dried uniformly, forming homogeneous films without visible phase separation or crystallization. In contrast, the urea/NaOH system (Figure 3c) exhibited non-uniform drying behavior accompanied by localized surface crystallization and slight wrinkling during solvent evaporation. The result indicates that the PEG and glycol ether suppressed the crystallization of the solution. Both co-solvents are hydrophilic and form hydrogen bonds with dissolved cellulose molecules. Moreover, the higher molecular weight of PEG than glycol ether prevents the aggregation of dissolved constituents of LCMFs or chemicals, due to the higher inherent viscosities of the LCMF solutions, higher than that of the urea/NaOH. Urea not only improves the dissolution yield but also influences the solution stabilizer, preventing the gelation [40,41,42,43]. When the water dried out, however, the formation of dried LCMF solutions indicates that urea and/or NaOH were exposed on the surface during drying, while the NaOH was not crystallized and was mixed with the glycol ether and PEG solution.

### 2.3. Regenerated LCMF Film Properties

**Figure 4:**
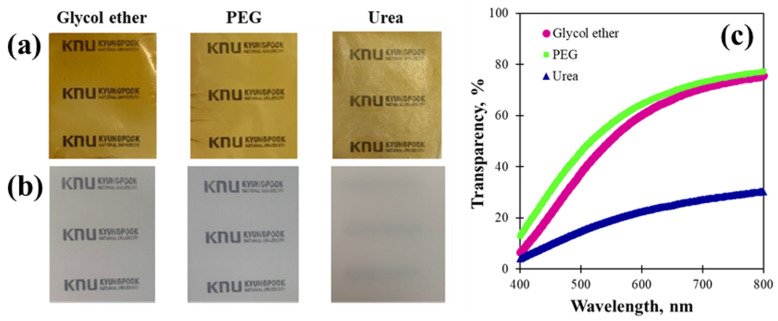
Transparency of the regenerated LCMF films: (**a**) visual appearance of the films placed on printed text; (**b**) visual appearance of the films positioned directly under the camera lens; (**c**) UV–visible transmittance spectra of the regenerated LCMF films.

As shown in Figure 2 and Figure 3, the LCMF dissolution form may differ depending on the dissolving solvent. Therefore, this difference in the regenerated form may affect the transparency of the prepared film. The optical properties of the LCMF films can be determined by evaluating the transparency to light. The visual observation and UV–vis transparency results of the regenerated LCMF films are shown in Figure 4. The regenerated films prepared with glycol ether/NaOH and PEG/NaOH solvents exhibited high transparency, even when placed farther from the object, as shown in Figure 4a,b. These results indicate that the transparency of the regenerated LCMF films might depend on the type of dissolution solvent used. This can be more clearly confirmed by changing the distance between the film and the object. As a result of these measurements, it was found that the film transparency was higher for the PEG/NaOH and glycol ether/NaOH solvents than for the urea/NaOH solvent. Unlike cellulose I, cellulose II is produced with an increased alignment. The dissolving solvent acts as a barrier between cellulose molecules in the modified form and controls the degree of hydrogen bonding in the modified form [44]. The lower transparency is attributed to microstructural changes during dissolution and regeneration, including partial recrystallization of cellulose, hemicellulose, and lignin in the urea/NaOH solvent system. These changes enhance light scattering in the regenerated films, indicating that the dissolving solvent affects both optical transparency and solution stability. These results thus suggest that the dissolved solvent affects both the transparency and the stability of the LCMF solution.

**Figure 5:**
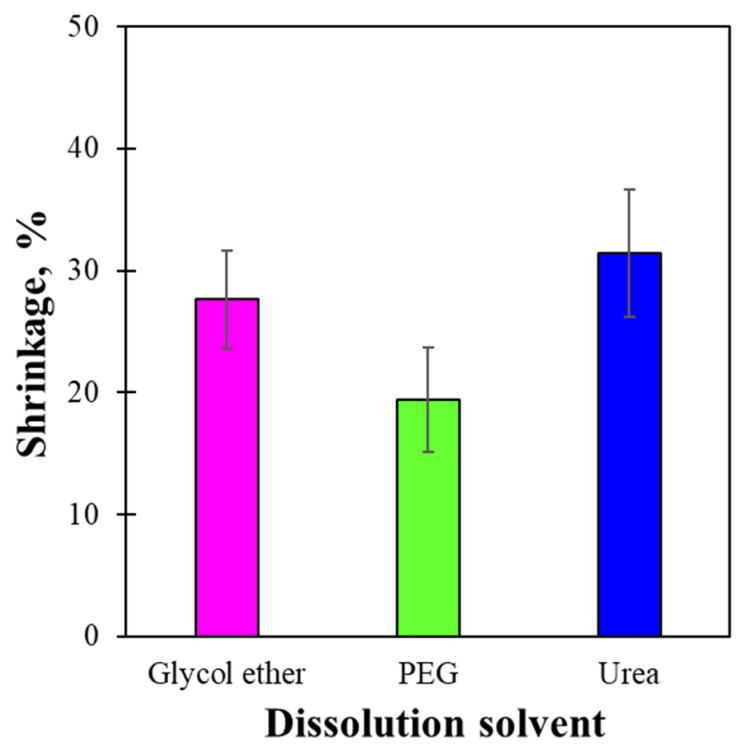
Effect of dissolution solvent on the shrinkage of regenerated LCMF films.

The shrinkage of the dried regenerated LCMF films was measured, as shown in Figure 5. The produced films were found to shrink during the regeneration process due to coagulation and gel drying, becoming smaller than their initial state. Primary shrinkage occurs during regeneration, whereas secondary shrinkage occurs during drying. No big difference was evinced between primary and secondary film shrinkage. The measured film shrinkages were 19.4%, 27.6%, and 31.4% for the PEG/NaOH, glycol ether/NaOH, and urea/NaOH solvents, respectively. As the LCMF component dissolved in the alkaline state and coagulated with 5% sulfuric acid, the dissolved glycol ether/NaOH, PEG/NaOH, and urea/NaOH solvents present in the LCMF solution were also removed. The hypothesis is that the time required for the polymeric glycol ether and PEG to escape from a 5% sulfuric acid aqueous solution is longer than that for low-molecular-weight urea. Thus, the lower shrinkage observed in the PEG/NaOH system may be attributed to delayed aggregation of dissolved LCMF molecules during coagulation, resulting in more gradual network consolidation and reduced internal stress development during drying.

**Figure 6:**
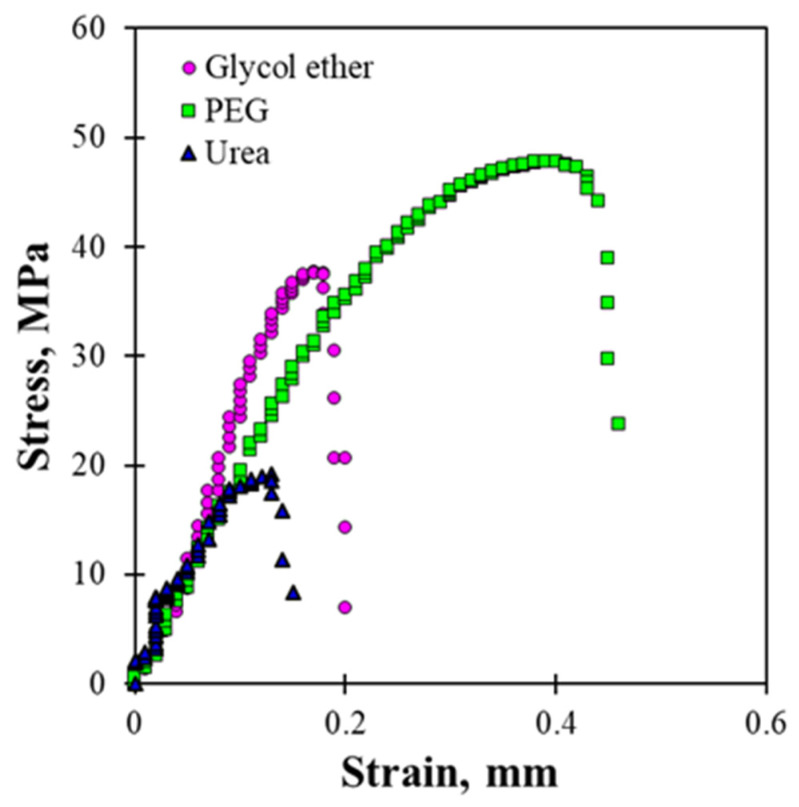
Stress–strain curve of the regenerated LCMF films.

The tensile stress–strain (S–S) curves of the regenerated LCMF films clearly show that the mechanical response varies depending on the dissolving solvent system (Figure 6). The film regenerated using the PEG/NaOH solvent exhibited the highest stress level throughout the deformation process, reaching a maximum tensile stress of 47.8 MPa. In comparison, the glycol ether/NaOH- and urea/NaOH-regenerated films showed lower maximum stresses of 37.6 MPa and 18.5 MPa, respectively. Differences in the strain range over which stress was sustained were also observed among the solvent systems, indicating distinct deformation behaviors in the S–S curves. The PEG/NaOH-regenerated film maintained higher stress values over a broader strain region, whereas the urea/NaOH-regenerated film exhibited early stress saturation and fracture. According to previous studies, the tensile performance of regenerated cellulose films is closely related to the compactness and structural organization of the regenerated network [45]. The S–S behavior observed for the PEG/NaOH system suggests the formation of a denser, more mechanically robust LCMF structure than in the other solvent systems. The superior tensile strength of the regenerated LCMF film prepared from the PEG/NaOH system is closely related to the higher solution stability observed in Figure 2. The higher inherent viscosity and the smallest viscosity reduction over storage time indicate that cellulose chains in the PEG/NaOH system maintain their molecular integrity more effectively, with reduced chain recombination during storage. This suggests improved retention of effective molecular weight and a higher degree of chain entanglement in the dissolved state [28]. During coagulation and regeneration, these well-preserved, highly entangled cellulose chains are more likely to reorganize into a continuous, compact network, thereby enhancing interchain interactions and stress-transfer efficiency within the regenerated film [7,15]. In contrast, the urea/NaOH system, despite its higher dissolution yield, exhibited a more pronounced viscosity decrease, suggesting partial chain scission or re-aggregation that limits the formation of a mechanically robust network. Notably, the highest dissolution yield did not correspond to the best final film performance. This suggests that dissolution rate alone does not govern mechanical or dimensional stability; rather, the stability of the dissolved chains and their ability to reorganize into a coherent network during regeneration appear to be more decisive factors. While dissolution yield and viscosity changes are directly supported by the data in this study, differences in chain rearrangement and network formation during the regeneration process were inferred based on dissolution yield and viscosity changes. Therefore, further insight into the underlying structural differences would require complementary structural characterization. Future work will involve additional structural analyses, including FT-IR and X-ray diffraction, to clarify solvent-induced structural changes in regenerated LCMF films.

## 3. Conclusions

This study investigated the dissolution behavior of LCMFs and the properties of regenerated films using glycol ether/NaOH, PEG/NaOH, and urea/NaOH solvent systems. Although the urea/NaOH system achieved the highest dissolution yield (85.0%), it led to partial recrystallization during drying, reducing film stability. In contrast, the PEG/NaOH system provided a favorable balance between dissolution efficiency, solution stability, and film performance, producing regenerated films with high transparency, low shrinkage (19.4%), and superior tensile strength (47.8 MPa). These results demonstrate that solvent selection plays a decisive role in controlling both dissolution behavior and final film quality, identifying PEG/NaOH as a promising solvent system for the fabrication of high-performance regenerated LCMF films.

## 4. Materials and Methods

### 4.1. Materials

Pine (*Pinus densiflora*) wood meals with a moisture content of 6.8% were ground into 40–80 mesh by a rotary speed mill (IKA MF10 basic, Staufen, Germany) and a mechanical lab sieving apparatus (Sieve shaker, CG-211-8, Chunggye, Seoul, Republic of Korea). The wood meals were extracted using a mixture of ethanol-benzene (1:2, *v*/*v*) for further chemical analysis. The Klason lignin and acid-soluble lignin contents of the extractive-free wood meal were 28.3% and 0.6%, respectively. All reagents and solvents of extra-pure grade, including glycol ether (134.17 g/mol), PEG (2000.00 g/mol), urea, NaOH (60.06 g/mol), sulfuric acid (H_2_SO_4_), ethanol, and benzene, were purchased from Daejung Chemicals (Siheung, Republic of Korea).

### 4.2. LCMF Preparation

The reaction temperature, time, and acid catalyst used to prepare LCMF samples as a feedstock for LCMF dissolution to fabricate regenerated LCMF films were selected based on previous studies [46]. The reaction time was 120 min, the liquor-to-wood meal ratio was 1:1.5 (*wt*/*wt*), and the acid catalyst was 3 vol%. After pulping, the residues were diluted 20 times with distilled water and then homogenized for 5 min at 2700 rpm in a laboratory homogenizer (HG-15A, DAIHAN Scientific Co., Ltd., Wonju, Republic of Korea). The micronized LCMF samples were sequentially washed with 0.5 N NaOH and 100% acetone. The washed sample was oven-dried overnight and then kept in a sealed bag for further experiments.

### 4.3. LCMF Dissolution

The dissolving solvents used in this study were prepared with different ratios of glycol ether, PEG, and urea, blended with a NaOH/water solvent, as shown in Table 4. The concentrations of PEG/NaOH [28] and urea/NaOH [47] were selected based on previous studies. The glycol ether/NaOH solvent was prepared with a composition of 1:9:90 wt% (glycol ether:NaOH:water), corresponding to the same NaOH concentration as that used in the PEG/NaOH dissolution solvent. All dissolution experiments were carried out with an LCMF content of 5% in each dissolving solvent. The mixture was dispersed using a magnetic stirrer for 3 h at room temperature and then subjected to a freeze–thaw process. The suspension was frozen at −15 °C for 12 h and subsequently thawed under vigorous shearing. This freeze–thaw procedure was repeated four times and adapted from previously reported NaOH-based aqueous systems for low-temperature dissolution [28,47]. After the final cycle, the resulting solution was centrifuged at 2500 rpm for 20 min (Centrifuge 1580, LABOGENE, Gimpo, Republic of Korea) to separate the dissolved and undissolved components. After washing the dissolving solvent and distilled water, the LCMF solution was freeze-dried (FD, IlShinBioBase Co., Ltd., Dongducheon, Republic of Korea).

### 4.4. Regenerated LCMF Films

**Figure 7:**
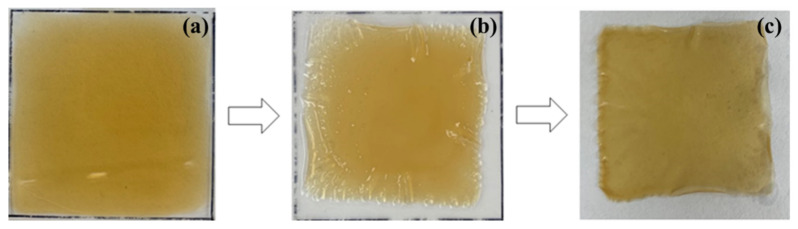
Regenerated LCMF process using the glycol ether/NaOH aqueous dissolution solvent; (**a**) LCMF solution casting; (**b**) LCMF coagulating in the 5% H_2_SO_4_ solution; (**c**) gel drying.

Regenerated films were prepared to investigate the physical properties of the regenerated dissolved component. The thickness of the prepared films was in the range of 15–25 µm. The dissolved component was transferred to a 50 µm glass plate, dried for 1–2 h, and then coagulated in a 5% sulfuric acid solution for about 5 min to form the regenerated film. The regenerated film was thoroughly washed by sedimentation in distilled water four times. After removing the water with filter paper, the regenerated film was dried at 45 °C in a Gel dryer (SE1160-230V, Pharmacla Biotech Inc., San Diego, CA, USA) for about 2 h, and was then left to relax in a desiccator at room temperature for 24 h. The overall regeneration procedure, including casting, coagulation, and gel drying, is illustrated in Figure 7. uring the drying and relaxation steps, no visible wrinkling of the films was observed.

### 4.5. Dissolution Yield

LCMF films were stirred with the dissolution solvents, then centrifuged twice to recover the dissolved (supernatant) and undissolved (residue) components. The insoluble residue was further washed twice by centrifugation with a 7 wt% NaOH aqueous solution to ensure complete removal of dissolved components, including any trapped cellulose or gel-like residues. The insoluble portion obtained through centrifugation was thoroughly washed two or more times with distilled water and then dried. The dissolution rate was calculated by measuring the weight of the dried insoluble part according to Equation (1):

(1) $$Dissolution\;yield(\%)=\frac{w_{0}-w_{1}}{w_{0}}\times 100$$

where *w*_0_ is the LCMF dried weight and *w*_1_ is the undissolved LCMF component.

### 4.6. Chemical Composition

In order to evaluate the behavior of cellulose, hemicellulose, and lignin content transferred to the LCMF-regenerated films, the neutral sugar of the undissolved component and the Klason lignin amount were determined using the TAPPI test method T222. The sugar composition was analyzed via the alditol-acetate method and calculated using the inositol peak area. The measurement was performed using gas chromatography (HP-6890, Agilent, Santa Clara, CA, USA) equipped with a silica capillary column (SP-2330, Supelco Inc., Bellefonte, PA, USA); detection was achieved through a flame ionization detector (FID). The nitrogen carrier gas had a flow rate of 45.0 mL/min. The initial temperature was 220 °C and was subsequently raised in steps of 2 °C to 240 °C.

### 4.7. Viscosity Measurement of the LCMF Solution Stability

The solvent mixtures, namely, glycol ether/NaOH, PEG/NaOH, and urea/NaOH, were prepared immediately before the viscometer measurement and maintained at 24 ± 0.1 °C. The LCMF solutions dissolved in glycol ether/NaOH, PEG/NaOH, and urea/NaOH were diluted with the corresponding solvent to a polymer concentration of approximately 4.0 × 10^−3^ g/mL for inherent viscosity measurements. The LCMF solution stability was evaluated by monitoring the inherent viscosity, calculated according to Equation (2), for each dissolution solvent over approximately 30 days [39].

(2) $$\eta_{\mathrm{inh}}=\frac{{In(\eta }_{r})}{c}$$

where η_inh_ is the inherent viscosity (dL/g), η_*r*_ is the relative viscosity defined as the ratio of the solution viscosity to the solvent viscosity, and *c* is the polymer concentration (g/mL).

### 4.8. Transparency of the LCMF-Regenerated Films

The UV transparency (400–600 nm) of the regenerated films was measured using specimens cut to 1.4 × 3 cm^2^ pieces, with transparency evaluated at 550 nm [48].

### 4.9. Shrinkage of the LCMF-Regenerated Films

The shrinkage of the films was calculated according to Equation (3):

(3) $$Shrinkage(\%)=\frac{a_{0}-a_{1}}{a_{0}}\times 100$$

where *a*_0_ is the fabricated film area, and *a*_1_ is the LCMF film area regenerated from the 5% H_2_SO_4_ aqueous solution during film production.

### 4.10. Tensile Strength of the LCMF-Regenerated Films

The thickness of the regenerated film was measured 10 times using a caliper (Daeil Machinery Co., Ltd., Cheonan, Republic of Korea), and the average thickness was then calculated. The specimens were cut into 3 × 1 cm^2^ pieces, and a universal tensile strength tester (OTT-00, OrientalTM Co., Ltd., Siheung, Republic of Korea) was used to measure the film tensile strength. The tester speed was set to 10 mm/min, and the gap between the clamps was set to 1 cm.

## Figures and Tables

**Table 1:** Composition of raw LCMFs.

Type	Raw Material			
	Total (g)	Carbohydrate (g)	Lignin (g)	C + L ^a^
LCMF	100.0	87.4	8.4	95.8

^a^ Sum of carbohydrate contents and lignin contents.

**Table 2:** Distribution of dissolved and undissolved LCMF components in different NaOH-based solvents.

Type	Solvent	Dissolved Component (Regeneration)				Undissolved Component (Residue)			
		Total (g) ^a^	Carbohydrate (g) ^b^	Lignin (g) ^c^	C + L ^d^	Total (g) ^a^	Carbohydrate (g) ^b^	Lignin (g) ^c^	C + L ^d^
LCMF	Glycol ether	76.2	67.7	3.5	71.2	23.8	18.9	4.0	22.9
	PEG	77.0	68.2	3.9	72.1	23.0	17.9	3.6	21.5
	Urea	85.0	71.6	5.2	76.8	15.0	12.0	2.3	14.3

^a^ Total dissolved fraction calculated by dissolution yield. ^b^ Total fraction calculated by dissolution yield and neutral sugar analysis. ^c^ Total fraction calculated by dissolution yield and Klason lignin contents. ^d^ Sum of carbohydrate contents and lignin contents.

**Table 3:** Composition of LCMF, dissolved, and undissolved components.

Sample		Klason Lignin, %	Neutral Sugar Analysis, %					
			Glucose	Arabinose	Xylose	Mannose	Galactose	Yield, % ^a^
Raw materials		8.4	95.9	<0.3	1.1	2.5	0.7	94.7
Glycol ether	Dissolved component	4.6	94.0	<0.3	0.9	3.1	1.7	88.9
	Undissolved component	16.7	96.5	<0.3	0.4	2.2	0.7	79.4
PEG	Dissolved component	5.0	94.7	<0.3	0.9	3.2	0.9	88.6
	Undissolved component	15.8	96.4	<0.3	0.4	2.3	0.8	77.9
Urea	Dissolved component	6.2	94.7	<0.3	0.9	3.1	0.9	84.2
	Undissolved component	15.5	96.0	<0.3	0.6	2.5	0.6	79.7

^a^ Total yield of the neutral sugar component.

**Table 4:** Preparation composition of the LCMF dissolution solvents.

Solvent	Composition Ratio, wt%					Reference
	Glycol Ether	PEG	Urea	NaOH	Water	
Glycol ether	1	-	-	9	90	This work
PEG	-	1	-	9	90	[27]
Urea	-	-	12	7	81	[46]

## Data Availability

The original contributions presented in this study are included in the article. Further inquiries can be directed to the corresponding author.

## Abbreviations

The following abbreviations are used in this manuscript:

CCUS	Carbon capture, utilization, and storage
CO_2_	Carbon dioxide
DP	Degree of polymerization
FD	Freeze-drying
FID	Flame ionization detector
GC	Gas chromatography
IPCC	Intergovernmental Panel on Climate Change
LCMFs	Lignocellulosic microfines
NaOH	Sodium hydroxide
PEG	Poly(ethylene glycol)
RC	Regenerated cellulose
UV	Ultraviolet
